# Supplementary material for: Pancreatic β Cells Inhibit Glucagon Secretion from α Cells: An In Vitro Demonstration of α–β Cell Interaction
Source: Nutrients. 2021 Jun 30;13(7):2281. doi: 10.3390/nu13072281 (PMC8308288; doi:10.3390/nu13072281)
Supplement: Supplementary file 1 [file nutrients-13-02281-s001.zip › nutrients-1275963-supplementary materails.pdf]

Supplementary Materials

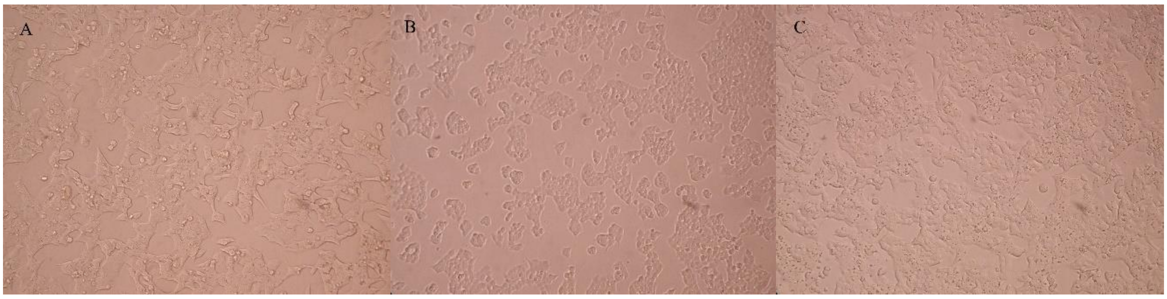

Figure S1. Morphology of MIN-6 cells (A), α-TC1-6 cells (B) and co-cultured cells (C) after 72 h incubation at 25 mM glucose. Optical microscope (X100).

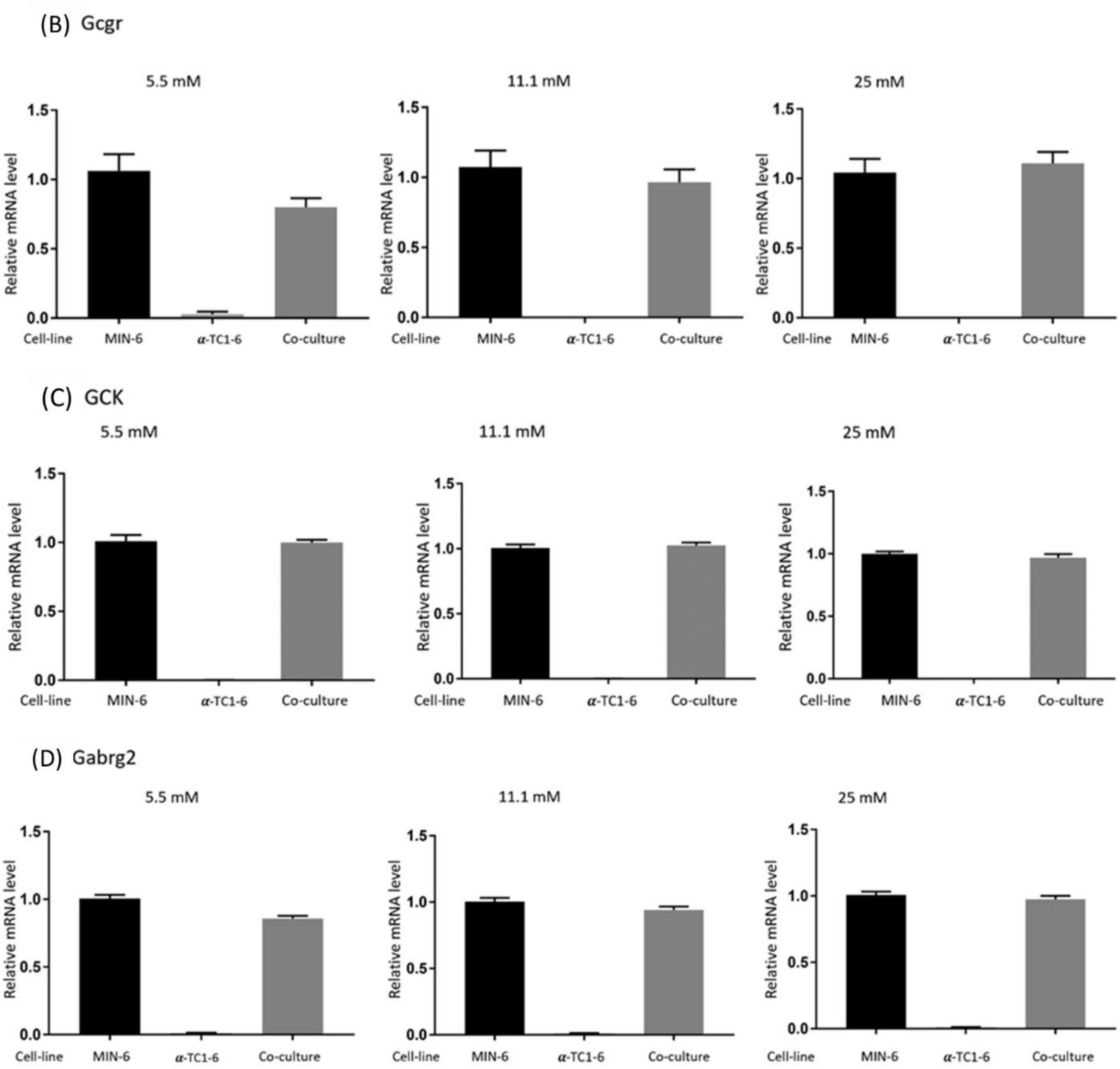

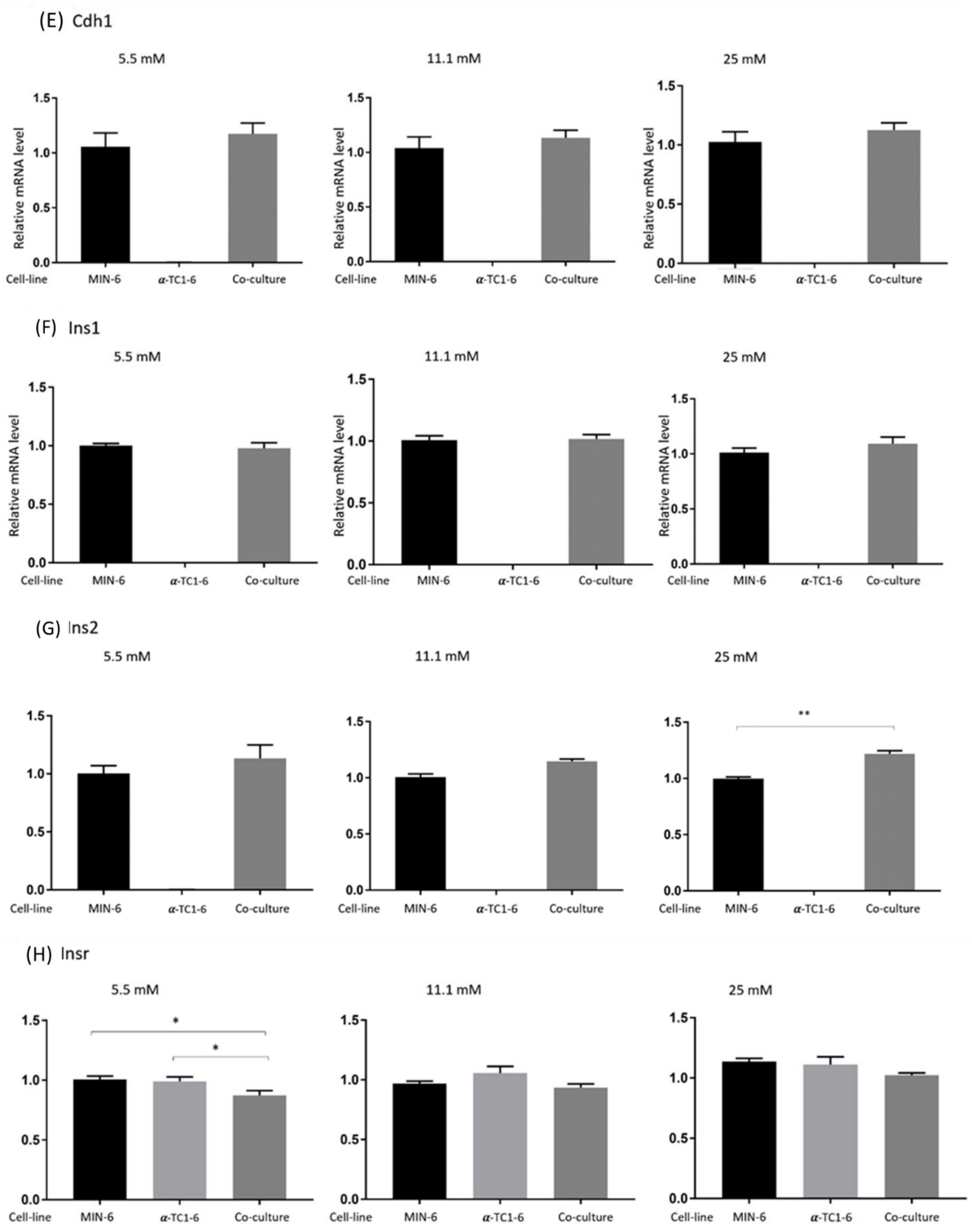
(E) Cdh1
5.5 mM
11.1 mM
25 mM
Relative mRNA level
1.5
1.0
0.5
0.0
Cell-line
MIN-6
α-TC1-6
Co-culture
(F) Ins1
5.5 mM
11.1 mM
25 mM
Relative mRNA level
Cell-line
MIN-6
α-TC1-6
Co-culture
(G) Ins2
5.5 mM
11.1 mM
25 mM
**
Cell-line
MIN-6
α-TC1-6
Co-culture
(H) Insr
5.5 mM
11.1 mM
25 mM
*
*
Cell-line
MIN-6
α-TC1-6
Co-culture

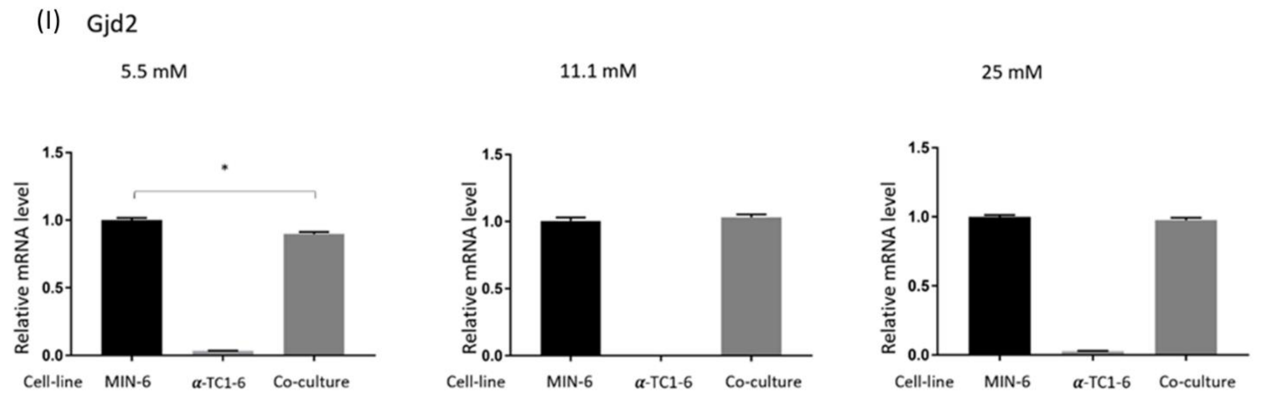

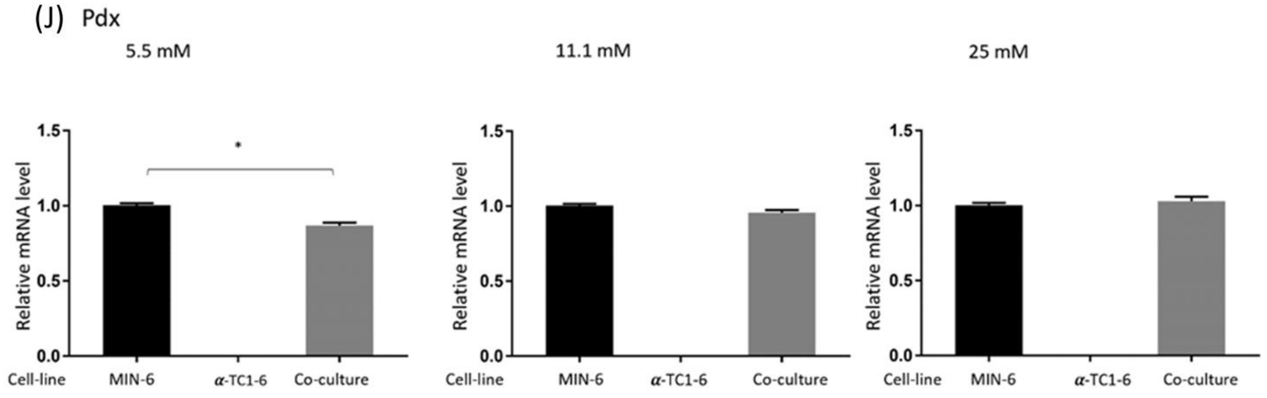

Figure S2. Effects on gene expression in mono- or co-cultured MIN-6 and α-TC1-6 cells after 72 h incubation at 5.5, 11.1 or 25 mM glucose. Transcript abundance of 20 specific genes was assessed by real-time RT-PCR using TaqMan assays, here shows the results of Gcgr, GCK, Gabrg2, Cdh1, Ins1, Ins2, Insr, Gjd2 and Pdx. Ten samples were obtained for each condition, and the samples were measured in quadruplicates. Data are presented as mean± SEM. *$p<0.05$, **$P<0.01$.
